# Active meta polarizer for terahertz frequencies

**DOI:** 10.1038/s41598-020-71990-z

**Published:** 2020-09-21

**Authors:** Hang Wong, Kai Xu Wang, Laure Huitema, Aurelian Crunteanu

**Affiliations:** 1 State Key Laboratory of Terahertz and Millimeter Waves, Department of Electrical Engineering, City University of Hong Kong, Tat Chee Avenue, Kowloon, Hong Kong; 2 XLIM Research Institute, CNRS /University of Limoges, 123 Avenue Albert Thomas, 87060 Limoges, France; 3 Harbin Institute of Technology, Shenzhen, China

**Keywords:** Electrical and electronic engineering, Applied physics

## Abstract

Active meta polarizers based on phase-change materials have recently led to emerging developments in terahertz devices and systems for imaging, security, and high-speed communications. Existing technologies of adaptive control of meta polarizers are limited to the complexity of external stimuli. Here, we introduce an active terahertz polarizer consisting of a single layer of large array patterns of vanadium dioxide material integrated with metallic patch matrix to dynamically reconfigure the polarization of the terahertz waves. The proposed active polarizer is simple in structure and can independently manipulate the polarization of the incident THz waves in two orthogonal directions. In addition, the device can also be performing as a highly efficient reflector at the same frequencies. We demonstrate that efficient and fast polarization changes of THz waves can be achieved over a wide operating bandwidth. Compared with other active polarizers using mechanical, optical and thermal controls, it can be conveniently manipulated with DC bias without any external actuators, intense laser source or heater. Therefore, with the advantages of high efficiency, compact size, low loss, low cost and fast response, the proposed polarizer can be highly integrative and practical to operate within adaptive terahertz circuits and systems.

## Introduction

Terahertz radiations, with their extraordinary advantages (large bandwidth, traveling in a line of sight, good penetration and non-ionizing) offer unprecedented capabilities in applications spanning from medical imaging^1^, security^2^, radar^3^, high-speed communications^4^ to spectroscopy of complex molecular networks^5^. In particular, active polarizers play an important role in terahertz imaging and wireless communication systems, which are essential for improving imaging and communication qualities^6,7^. Recent technologies of adaptive control of propagating terahertz wave polarizations are based on meta-devices (collection of coupled identical meta-atoms) which modify their intrinsic overall topology through mechanical, optical, electrical and thermal stimuli^8–33^.

Microelectromechanical system (MEMS) approaches have been used to enable the dynamic control of the chiral properties of metamaterials devices^8–10^. In these implementations, the suspension angle of a cantilever in air can be precisely tuned through electrostatic^8^ or thermal^9^ actuations, which induce the modification of polarization states of an incident THz radiation. In addition, the electromechanically controlled anisotropy of THz radiation is manipulated by a comb-drive actuator^11^. Besides, a handedness-switchable chiral metamaterial for polarization modulation can also be produced by a pneumatic force^12^. However, the required external actuators have limited switching speeds, which may not be fast enough for terahertz applications.

Optical photoexcitation modifying the surface and bulk conductivity of semiconductor substrates through photocarrier generation is effective to realize THz polarizers^13,14^. Thus, the optical stimulation of metamaterial devices made of bilayer metallic patterns (counter-facing gammadion shaped resonators with different sizes) on a semiconductor substrate allowed terahertz polarization rotation angles as large as 45°^14^. In addition, the semiconductor material (epitaxial Si patterns) can also be embedded in metallic chiral resonators^15–17^, and can function as an optical switch controlling a specific state of the polarizer. Different other optical approaches use shadow masks^18^ or programable photo patterns^19^ to project intense light on Si substrates for manipulating the polarization of the THz incident wave. Although these approaches show a higher degree of flexibility, they require external intense laser sources, which are increasing the size of the overall polarizer device.

Electrical controls are also used to manipulate the polarization of terahertz radiation. Liquid crystals (LCs) are good candidates for electrically manipulated active materials at THz frequencies using metallic^20^ or porous graphene electrodes^21^. Thus, a reflective terahertz waveplate based on LC layers embedded with a metal wire grid is used to control the LC refractive index^20^. By tuning the applied voltage, the THz incident wave polarization can be appropriately converted. Apart from LCs, graphene is another promising material for electrically tunable THz waveplates^22^. An active polarizer with two layers of graphene^23^ shows that the transmission of a terahertz wave can be electrically modulated to convert the polarization of the reflected wave. In these designs, although no external actuator or laser source is required, multilayers including DC-bias layers are necessary to control the LC or graphene, which result in high transmission losses and complicated fabrication.

Thermal stimuli can be conveniently applied to the manipulation of the response of terahertz devices with integrated phase transition materials like vanadium dioxide (VO_2_)^24–28^. The potential of using VO_2_ integration for realizing THz agile metamaterials comes from its aptitude to perform a reversible metal–insulator transition (MIT) spanning large frequencies domains, from DC to microwaves up to THz and near infra-red frequencies^24,2728^. The MIT is accompanied by large and abrupt changes in the material’s electrical and optical properties (e.g. up to 5 orders of magnitude change in electrical conductivity between the two states) and can be triggered not only by temperature but also electrically or optically^29,30^. One of the most remarkable characteristics of MIT in VO_2_ is the broadband response of the transition manifested by drastic electrical and dielectric properties changes between the insulator and metallic states^24–33^ and consequently, its integration potential for high-frequency applications such as broadband THz switches and modulators^24,25^, reconfigurable filters^26–28^ or millimeter-waves antennas^34^.

Specifically, a THz polarizer device with a VO_2_ layer placed underneath metallic gratings is able to switch the polarization state of the active device through heating^35^. The VO_2_ can also be integrated with metamaterials^36,37^ to manipulate the polarization of THz waves. A cross-shaped array with VO_2_ pads is proposed^36^ to convert an incident wave from linear polarization (LP) to circular polarization (CP). A similar thermally-active polarizer used VO_2_ films on E-shaped resonators can successfully enable the conversion^37^ of wave polarizations. The above-mentioned designs require external heaters to modify the state of the phase transition materials integrated in the metamaterials topology, which result in high energy costs, complex device structures and poor localization of thermal distribution.

Here, we are reporting on a new approach to achieve an active reconfigurable THz polarizer by exploiting an electrically induced current in VO_2_ pads to generate Joule heat, producing an insulator-to-metal transition (IMT) in the material. A single layer polarizer is designed and is fabricated on a c-type sapphire (Al_2_O_3_) substrate. The device consists of a large periodic VO_2_ patterns array co-integrated with a matrix of metallic patches, which are acting as static elements of the polarizer and, at the same time, are conveying (through lateral electrode connections) the polarization bias for electrically modifying the state (insulator or metallic) of the VO_2_ material patterns. The two-dimensional array of alternating VO_2_-metal patches allows, by applying a polarization voltage on the lateral electrodes (in *x-* or *y-*direction), to modify the topology of the device (generation of a grating-type topology in *x-* or *y-*direction) and induce a linear polarization of the transmitted THz radiation in the corresponding direction. Thus, within the same device, the voltage activation at the metallic state of VO_2_ pattern arrays in orthogonal directions allows to prepare a specific linear polarization state of the initially un-polarized incident THz radiation. Moreover, when electrically activating the VO_2_ patterns in both *x-* and *y-*directions, the device will behave as a highly efficient reflector for the incident THz waves in the 300–400 GHz domain.

## Results

### Tunable active polarizer with vanadium dioxide

**Figure 1:**
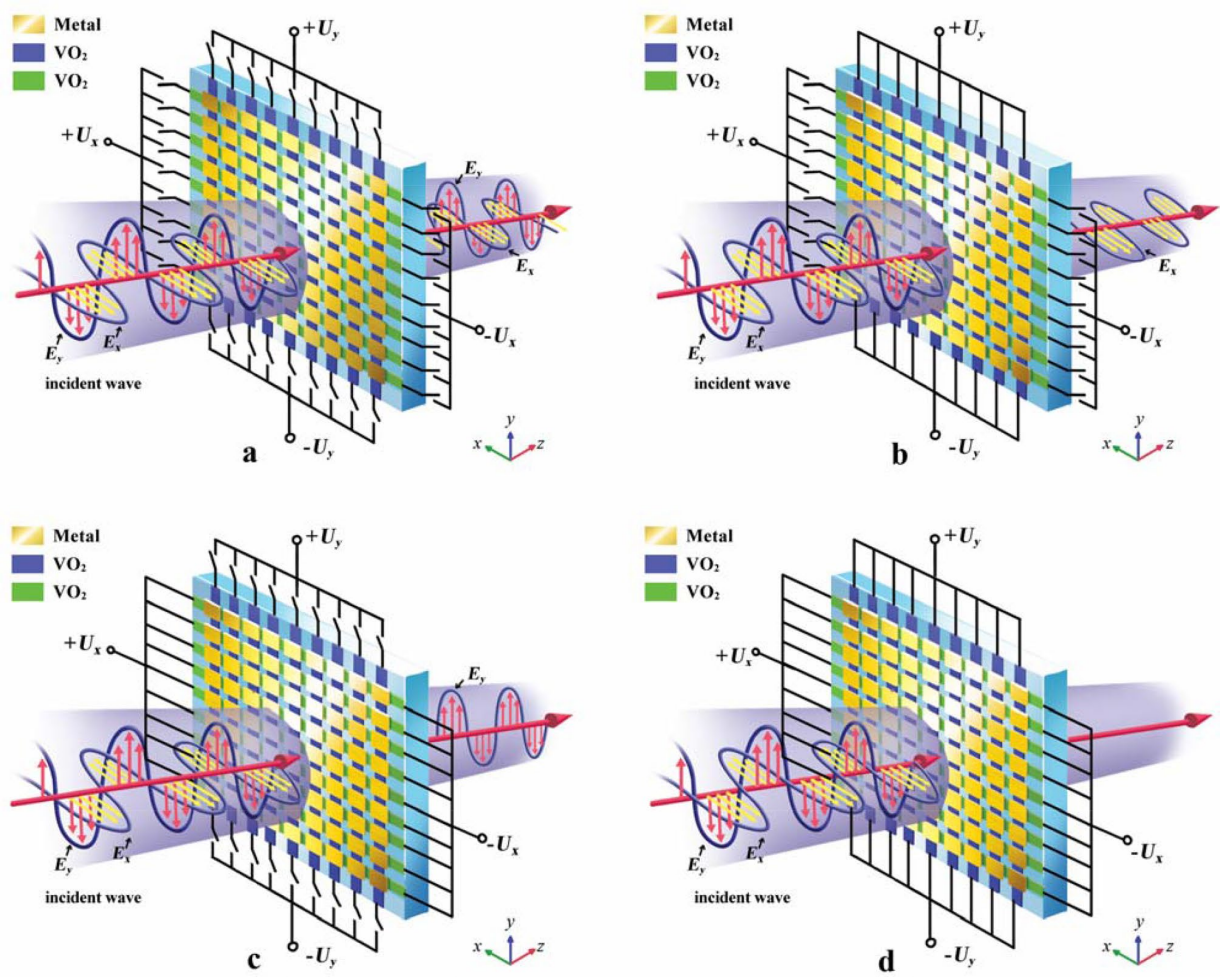
Designs of the electrically driven polarizer with VO_2_ patterns. (**a**) Polarizer without external voltage stimulus. As all VO_2_ are in the insulating state (not interfering with the incident radiation), *x*-and *y*-polarized incident waves can pass through the polarizer. (**b**) Polarizer under an external stimulus of *U*_*y*_*.* Blue VO_2_ patterns (on *y*-direction) are transformed to metallic state under *U*_*y*_ and the *y*-polarized incident wave is blocked. (**c**) Polarizer under an external stimulus of *U*_*x*_*.* Green VO_2_ patterns (on *x*-direction) are electrically activated to their metallic state and the *x*-polarized incident wave is blocked. (**d**) Polarizer under external stimuluses of *U*_*x*_ and *U*_*y*_*.* Both green and blue VO_2_ patterns are excited to their metallic state, consequently, *x*- and *y*-polarized incident waves can be blocked.

The topology of the active polarizer device as illustrated in Fig. 1 consists of a matrix of metallic patches (gold-colored patterns), VO_2_ patches arranged in the vertical direction (*y*-direction, blue-colored patterns) and VO_2_ patches arranged in the horizontal direction (*x*-direction, green-colored patterns). In both *x-* and *y*-directions, the VO_2_ elements are connected via the metallic patterns to external electrodes on which the applied bias voltage is used to trigger the metal–insulator transition of the VO_2_ patterns in a specific direction. When a voltage *U*_*x*_, or respectively, *U*_*y*_ (having values higher than 130 V, the threshold voltage needed to trigger the meta-insulator transition in the VO_2_ patterns) is applied on the metallic electrodes, the VO_2_ patterns in the specific triggered direction can switch from their insulating state (with a conductivity of 10 S/m) into their metallic state (conductivity of ~ 3.2 × 10^5^ S/m), which can be used to control the state of the wave polarization in the desired direction. When no voltage is applied (Fig. 1a), the polarizer can be view as a transparent surface to both *x-* and *y-*polarized incident waves. However, when the voltage *U*_*y*_ is applied (Fig. 1b), the polarizer can block the *y*-polarized incident wave, and the *x*-polarized incident wave can pass through. While the voltage *U*_*x*_ is applied, the device can block the *x*-polarized incident wave and transmit the *y*-polarized incident wave (Fig. 1c). When the voltages *U*_*y*_ and *U*_*x*_ are applied simultaneously, the polarizer acts as a reflector (Fig. 1d) to block all the incident THz wave.

### Circuit model and transmission coefficient

**Figure 2:**
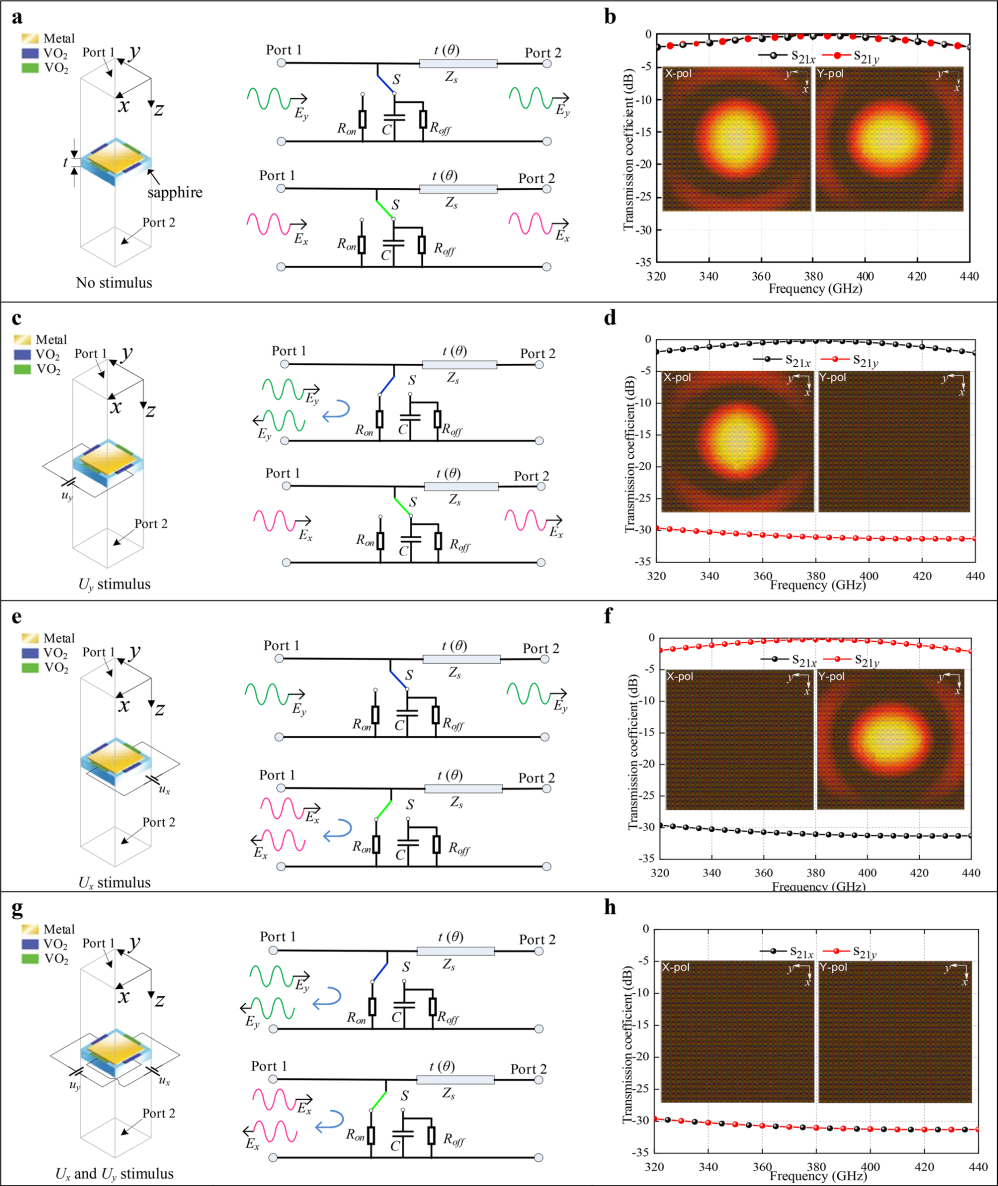
Circuit models and transmission coefficients. (**a**) Schematic models of polarizer unit for *x*- and *y*-polarized incident waves without external voltage stimulus. (**b**) The transmission coefficients for *x*- and *y*-polarized incident waves without external voltage stimulus. (**c**) Schematic models of polarizer unit for *x*- and *y*-polarized incident waves with stimulus of *U*_*y*_. (**d**) The transmission coefficients for *x*- and *y*-polarized incident waves with stimulus of *U*_*y*_. (**e**) Schematic models of polarizer unit for *x*- and *y*-polarized incident waves with stimulus of *U*_*x*_. (**f**) The transmission coefficients for *x*- and *y*-polarized incident waves with stimulus of *U*_*x*_. (**g**) Schematic models of polarizer unit for *x*- and *y*-polarized incident waves with stimuluses of *U*_*x*_ and *U*_*y*_. (**h**) The transmission coefficients for *x*- and *y*-polarized incident waves with stimuluses of *U*_*x*_ and *U*_*y*_.

Figure 2 represents, for all configurations presented in Fig. 1, the 3D electromagnetic model of the unit cell of the polarizer (metal and VO_2_ patterns deposited on a 100-µm thick c-cut sapphire substrate.), its equivalent circuit model and the associated simulated responses of the device, as transmission S_21_ parameters in both *x*- and *y*-directions. The polarizer is designed as a matrix of 30 × 30 square-shaped metallic elements (40 × 40 µm^2^) separated by 10 µm and connected through 10-µm length by 20-µm width VO_2_ patterns in both *x*- and *y*-directions, with an overall active area of 1.5 × 1.5 mm^2^. Besides their role of conveying the bias to the VO_2_ patterns, the metal patches also perform as an anti-reflective layer to help reduce the reflection loss of the polarizer. The equivalent circuits in *x*- and *y*-directions associated with different states of the device were used to underlie the mechanism responsible for the chirality switching in the terahertz active polarizer which can manipulate the *x*- and *y*-polarized incident waves independently. In the equivalent circuit of the metamolecule (Fig. 2a), the sapphire substrate can be modeled as a transmission line while the metal patch and the associated VO_2_ patterns are equivalent to an electrically-controlled switch *S* having two states: the first state, when the VO_2_ patterns are insulating, can be seen as a capacitance *C* in parallel with a high *R*_*off*_ resistance, whereas the second state, when the VO_2_ is electrically transformed to its metallic state, can be modeled by a low *R*_*on*_ resistance. If no DC voltage is applied over the VO_2_ patterns, the switch *S* connects to the capacitor *C* and the resistor *R*_*off*_. Both *x*- and *y*-polarized incident waves can pass through the active polarizer with an isotropic equivalent circuit due to the symmetrical structure of the polarizer. The polarizer has the same transmission coefficients (Fig. 2b) for both *x*- and *y*-polarized incident waves, when no DC voltage is applied. The transmission coefficients are closed to 0 dB at the center frequency of 380 GHz, which implies that the incident waves are completely transmitted through the polarizer. The inset figures show the transmitter power of *x*- an *y*-polarized wave. It can be found that both of them can easily pass through the polarizer.

Under applied DC voltages *U*_*x*_ or *U*_*y*_ -higher than the threshold voltage of the MIT, the VO_2_ elements will transform from their insulating to their conducting states (see supplementary material Figs. S3 and S5). When voltage *U*_*y*_ is applied in the vertical direction, the blue VO_2_ pads are activated to the metallic state (Fig. 2c) and the green VO_2_ pads remain insulating. Accordingly, the equivalent circuit for *x*- and *y*-polarized incident wave are different. For the *y*-polarized wave, the switch connects in this case to the resistor *R*_*on*_, corresponding to the conductive VO_2_ patches. While, for the *x*-polarized incident wave, the switch *S* is connecting to the capacitor *C* in parallel with the resistor *R*_*off*_. As a result, the *y*-polarized incident wave is blocked but the *x*-polarized wave can be transmitted through the polarizer. The simulated results of the S_21_ transmission coefficients and electric field distributions corresponding to this case (Fig. 2d) for *x*- and *y*-polarized incident waves, validate our analysis. Similarly, when the voltage *U*_*x*_ is applied in the horizontal direction, the green VO_2_ patterns turn into their conductive state and the blue VO_2_ elements keep their insulating state (Fig. 2e). The equivalent circuit in Fig. 2e is opposite to that in Fig. 2c. Therefore, the *x*-polarized incident wave is blocked and the *y*-polarized wave can pass through (Fig. 2f) the polarizer. Finally, when both *U*_*x*_ and *U*_*y*_ are applied simultaneously (Fig. 2g), VO_2_ patterns in both *x*- and *y*-directions are activated to their metallic state. The switches in the equivalent circuits are connected to the resistors *R*_*on*_ (Fig. 2g) in both directions, and both *x*- and *y*-polarized waves have transmission S_21_ parameters lower than − 30 dB, as shown in Fig. 2h. In this case, the device is behaving as a high-performance reflector.

### Electromagnetic performances

**Figure 3:**
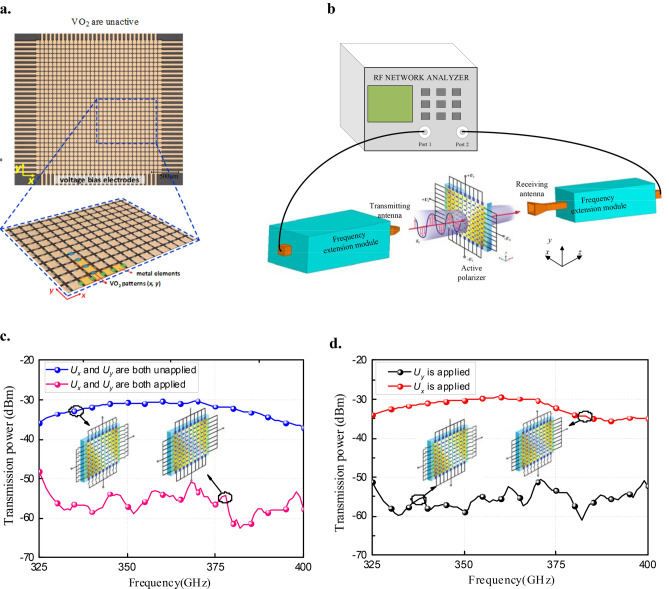
Measurement of an electrically driven polarizer with VO_2_ patterns. (**a**) The photos of a prototype of the active polarizer with VO_2_ patterns array co-integrated with a matrix of metallic patches. (**b**) Measurement set-up used for performance validation of the active polarizer for a transmitted power of the *y*-polarized incident wave between two THz antennas. (**c**) Measured transmission power between the two THz antennas with/without applied biases *U*_*x*_ and *U*_*y*_ on the active polarizer. (**d**) Measured transmission power between two THz antennas with an applied bias either *U*_*x*_ or *U*_*y*_ on the active polarizer.

The active area of the fabricated THz polarizer device is represented in Fig. 3a along with a zoomed image allowing to distinguish the metallic elements and the corresponding VO_2_ patterns in both *x*- and *y*-directions. The overall device topology along with the electrical and thermal activation properties of VO_2_ patterns in both directions are detailed in the supplementary material (and associated Figs. S1–S3).

The polarization performance of the integrated VO_2_ polarizer device was evaluated using the set-up presented in Fig. 3b based on a network analyzer, two terahertz extension modules, a DC power controller and a transmitting and a receiving antenna. The polarizer is placed vertically between the transmitting receiving antennas. Apertures of the transmitting and the receiving antennas are aligned face-to-face and with the same polarization. The measurements of the fabricated prototype were performed using *y*-polarized incident waves (We used an in-house designed waveguide aperture with the size of 0.508 mm × 0.254 mm and a horn antenna as the transmitting antenna and receiving antenna respectively. The active area of the polarizer is large enough for covering the aperture of the transmitting antenna. When we put the polarizer closely in front of the transmitting antenna, the polarizer can completely cover the beam spot.). When no DC bias voltage is applied to the polarizer, the *y*-polarized incident wave can pass through the polarizer (Fig. 3c) from the transmitting antenna to the receiving antenna. The measured transmitted power (blue color in Fig. 3c) is -30 dBm. When both *U*_*x*_ and *U*_*y*_ bias voltages (higher than the MIT threshold voltages of the VO_2_ patterns) are applied, the incident waves are blocked, resulting in transmitted power levels less than -50 dBm (red curve in Fig. 3c). Since the incident wave is polarized in the *y*-direction, it cannot pass through the polarizer if the *U*_*y*_ bias voltage is applied (black color curve in Fig. 3d); while it can still pass through the device if the *U*_*x*_ bias voltage is applied to the VO_2_ patterns oriented in this direction (red color curve in Fig. 3d). The average extinction ratio between the two extreme cases is around − 25 dB and the extinction ratio (> 20 dB) bandwidth is around 16% from 330 to 390 GHz. The roll off phenomenon in the result displayed on Fig. 3c,d was due to the response of the power amplifier used in the experiment. The amplitude of the power amplifier in the experiment has an output drop at and beyond the frequency of 380 GHz. As the polarizer has a symmetrical structure, the responses of *x*-polarized incident wave are similar to the results of the *y*-polarized incident wave. These results indicate that the polarizer can act as a transparent surface or an opaque surface to both *x-* and *y*-polarized incident waves, depending on the states (insulating or metallic) of the VO_2_ patterns array. By selectively controlling the *U*_*y*_ and *U*_*x*_ activation voltages, the polarizer can operate as a polarized-selective surface in both directions while having a single layer structure.

**Figure 4:**
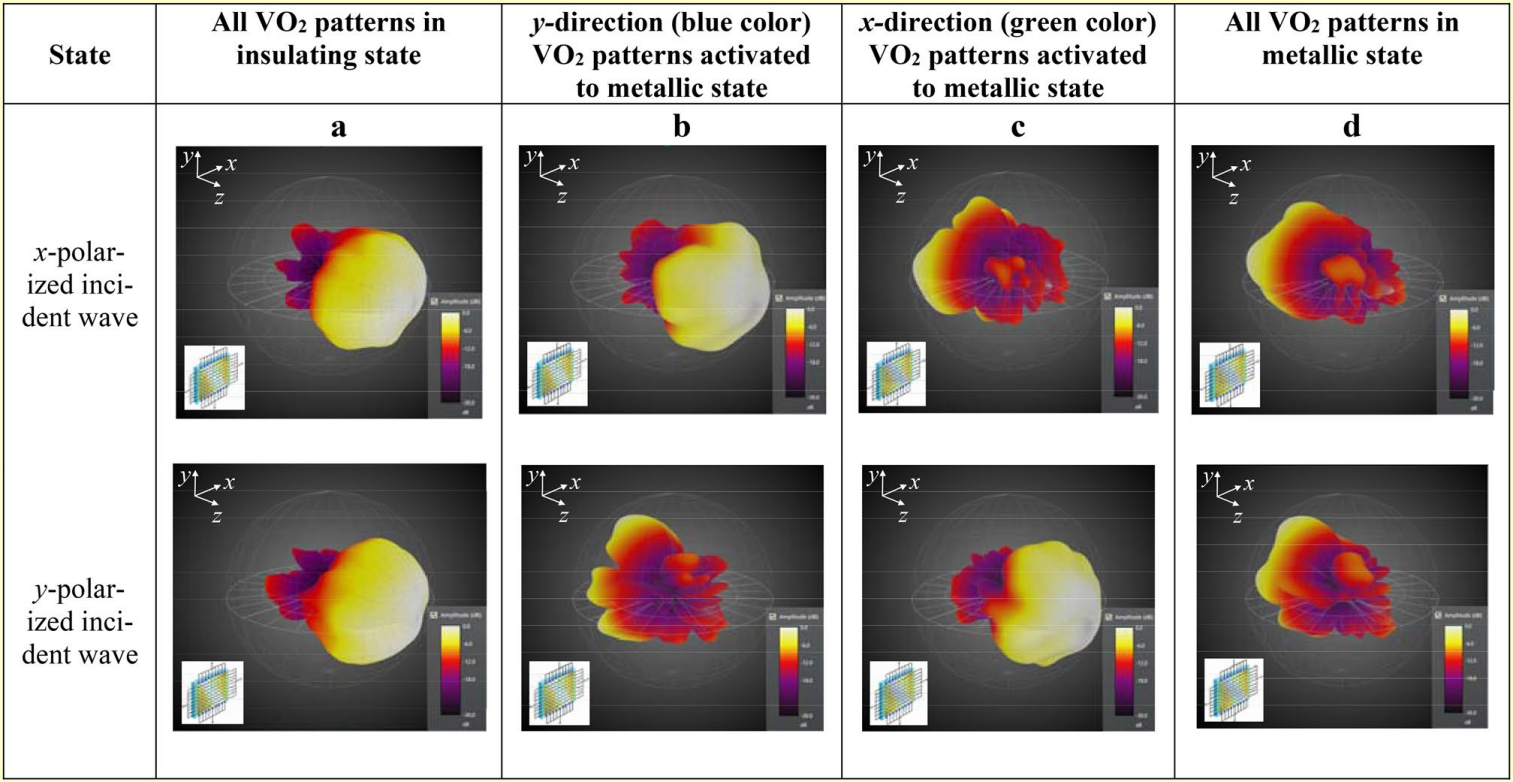
Far-field radiation patterns of the transmitted THz radiation through the active polarizer for different states of the VO_2_ patterns. (**a**) Both *x*-polarized and *y*-polarized incident waves can pass through the active polarizer when all VO_2_ patterns are in the insulating state. (**b**) Only *x*-polarized wave can pass though the active polarizer when *y*-direction (blue-color) VO_2_ patterns are activated to metallic state. (**c**) Only *y*-polarized wave can pass though the active polarizer when *x*-direction (green-color) VO_2_ patterns are activated to metallic state. (**d**) Both *x*-polarized and *y*-polarized incident waves are reflected by the active polarizer when all VO_2_ patterns are in metallic state.

The simulated far-field radiation patterns of the THz waves transmitted through the polarizer confirm the functionality of the integrated device at terahertz frequencies (Fig. 4). From the different states of the functional device activated by alternatively *U*_*x*_ and *U*_*y*_ applied voltages, it can be found that the maximum radiation direction for both *x*-and *y*-polarized incident waves are along *z* direction. When *U*_*y*_ is applied (blue-color type VO_2_ patterns in the *y*-direction are turned to their metallic state as in Fig. 1b), the *x*-polarized radiation pattern is not changing but the *y*-polarized incident waves are blocked. On the contrary, when *U*_*x*_ is applied (green-color type VO_2_ are turned to their metallic state as in Fig. 1c), the radiation pattern having *x*-polarization is highly degraded while the *y*-polarized radiation pattern is unaffected. Finally, if all the VO_2_ patterns (in both *x*- and *y*-directions) are turned to their metallic states, both *x*- and *y*-polarized radiation patterns are mostly reflected.

### Current and thermal distribution within the device

**Figure 5:**
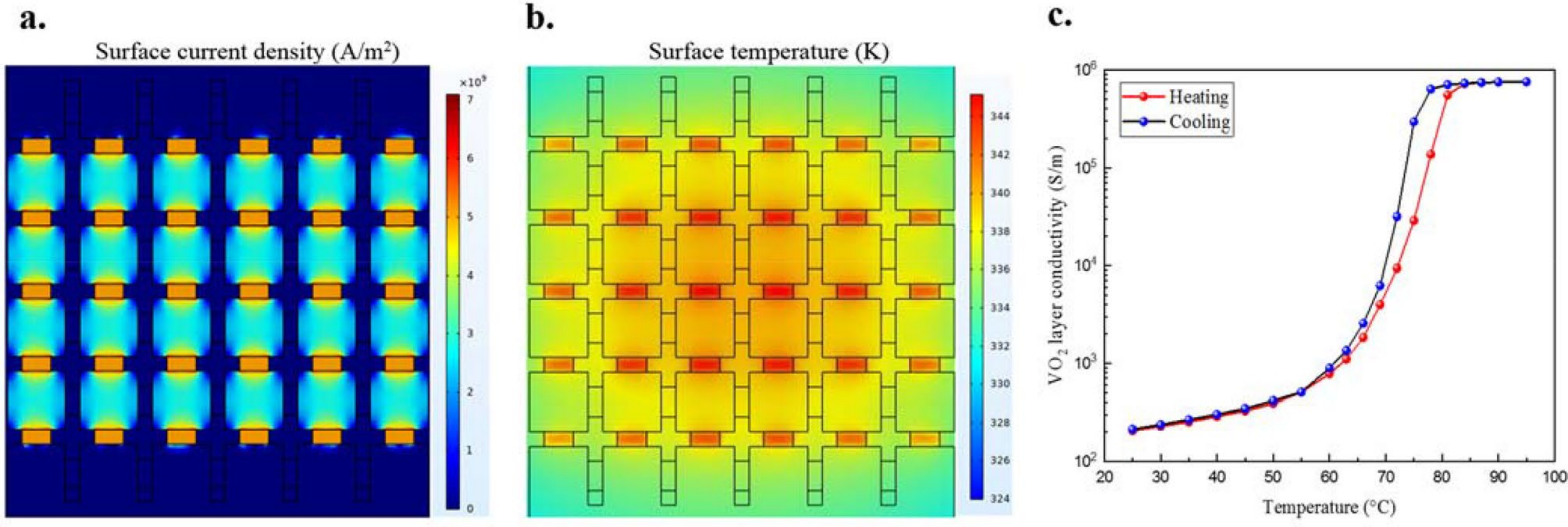
Current distribution and thermal distribution of the active polarizer when DC bias is applied. (**a**) Current distribution on the metallic and the VO_2_ pads when *U*_*y*_ is applied (currents only pass through the vertical VO_2_ pads). (**b**) Thermal distribution of the active polarizer when *U*_*y*_ is applied (currents can heat the vertical VO_2_ pads without affecting the horizontal VO_2_ pads). (**c**) VO_2_ conductivity versus temperature. When the temperature reaches to 344 K (71° C), the VO_2_ patches can switch from insulating state into metallic state.

Phase-change materials are sensitive to temperature. Although we use electrical stimuli to activate the conductive state of VO_2_ patterns of the polarizer, electrical currents going through the VO_2_ will result in heat dissipation and may alter the state of the VO_2_ elements in the opposite direction. To demonstrate that the thermal distribution (effectively controlled by the electrical stimuli), is confined only in the desired operation direction of the device, we performed electro-thermal simulations of the device’s behavior using finite element modelling (FEM). A relationship between the currents and thermal distributions in the *y*-direction of the active polarizer is represented in Fig. 5a,b. As only vertical currents (in the *y-*direction) flow through the VO_2_ patterns, their surface temperature is progressively increased (Fig. 5b). When the temperature dissipated in the VO_2_ patterns is higher than 344 K (71 °C), they are switching from an insulating state to a metallic state (Fig. 5c). However, the VO_2_ patterns in the orthogonal direction (in the *x-*direction) remain in their insulating state (Fig. 5b). These results confirm that the electrical activation approach is effective to trigger the conductive state of VO_2_ patterns in a specific direction, without modifying the state of the material in the opposite direction. Thus, the applied voltage can be independently applied to horizontal and vertical directions integrating the VO_2_ elements, fulfilling the desired function of the THz active polarizer device.

## Discussion

In conclusion, we have demonstrated a convenient method to activate a large VO_2_ array pattern integrated within a metallic elements matrix by using electrical stimuli. The approach provides different specific functionalities for a terahertz active polarizer which can be controlled using voltage bias in *x*- and *y*-directions. Moreover, the active polarizer demonstrated in this work is effective to generate high purity *x*- or *y*-polarized waves by appropriate selections of the VO_2_ patterns activation between their insulating and conductive states. Our approach will allow introducing versatile, rapid and highly-efficient active terahertz devices operating with a high degree of integration for future adaptive THz systems (modulators, reconfigurable metasurfaces, beam steering planer lens).

## Methods

### Device fabrication

The polarizer device was fabricated in a cleanroom environment by standard photolithography and deposition procedures using a two-mask levels process. Firstly, a 200-nm thick VO2 layer have been obtained on 100-µm thick c-cut sapphire substrate using reactive electron-beam evaporation of a vanadium target in an oxygen atmosphere^31,32^. The VO_2_ elements were patterned using a lithographically-defined photo-resist mask and a wet etching process. The subsequently deposited metallic patterns (40/1,000-nm thick Ti/Au bilayers) spanning the VO_2_ patches were obtained by electron-beam evaporation of the respective metallic elements and an optical lithography step using the lift-off method.

### Simulation methodology

The initial dimensions of the polarizers include the period and fill factor can be selected according to reference^38^. Then a full-wave simulation was performed using the commercially available Ansys HFSS to optimize its performance. In the initial simulations, the VO_2_ material were modeled using perfect E plane to reduce the computational burden during the design process but the actual structure was included in the final full-wave simulations. Joule heating simulations were performed using the COMSOL Multiphysics. The sapphire substrate we used in this experiment and in the associated electromagnetic and multi-physics simulations is a c-cut Al_2_O_3_ with anisotropic electromagnetic and thermal properties. It has a permittivity of *ε*_*x*_ = 9.3, *ε*_*y*_ = 9.3 (within the substrate surface plane, perpendicular to C-axis) and *ε*_*z*_ = 11.5 (parallel with C-axis ) and a loss tangent of 10^–4^. For the thermal properties used in the multi-physics simulations, we used thermal conductivities (at 300 K) of 25 W/(m*x*K) for the direction perpendicular to the C-axis and of 23 W/(m*x*K) for the direction parallel to the C-axis.

### Measurement methodology

To verify the design experimentally, a setup is built for far-field measurements, as shown in Fig. 3b. A pair of THz module (OML V02.2VNA2-T/R) is connected to the vector network analyzer (Agilent N5245A) to extend the operating frequency to 325–500 GHz. The transmitting antenna is an open waveguide which can generate a vertically polarized EM wave. While the receiving antenna is a standard horn, which has the same polarization with the transmitting antenna. The testing sample was inserted between the transmitting antenna and receiving antenna.

## Supplementary information

Supplementary Information.

## Data Availability

All data needed to evaluate the conclusions in the paper are present in the paper and/or the Supplementary Materials. Additional data related to this paper may be requested from the authors.
