# Supplementary material for: Active meta polarizer for terahertz frequencies
Source: Sci Rep. 2020 Sep 21;10:15382. doi: 10.1038/s41598-020-71990-z (PMC7506558; doi:10.1038/s41598-020-71990-z)
Supplement: Supplementary file 1 — Supplementary Information. [file 41598_2020_71990_MOESM1_ESM.docx]

**Supplementary information for:**

**Active Meta Polarizer for Terahertz Frequencies**

Hang Wong, Kai Xu Wang, Laure Huitema, and Aurelian Crunteanu


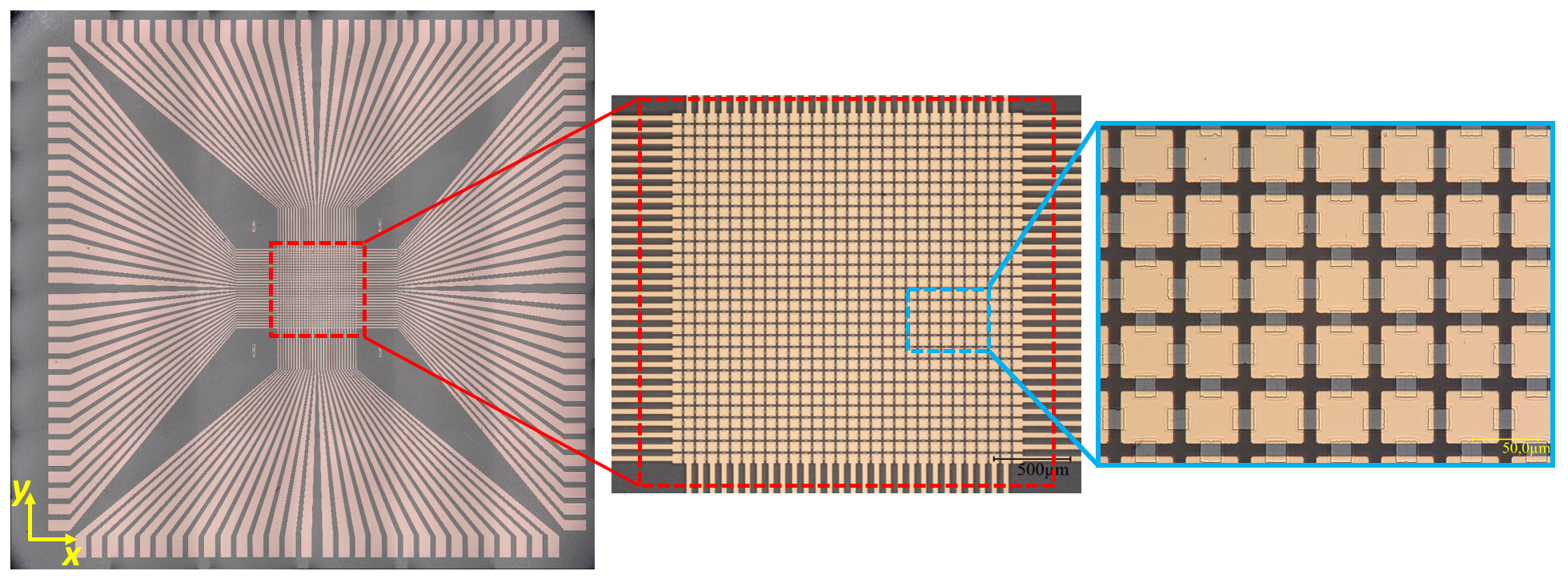


**Supplementary Figure S1 The image of the fabricated polarizer highlighting the 1.5×1.5 mm^2^ active area and details of the arrangements of the VO_2_ and metallic patterns in *X* and *Y* directions**. The polarizer is located at the center of the whole structure. The separated DC bias electrodes are used to excite the VO_2_ pattern independently. It can be found that some part of the VO_2_ pads are underneath the metallic patches, which cannot influence the behavior of the device since they are “shadowed” by the metallic patches.


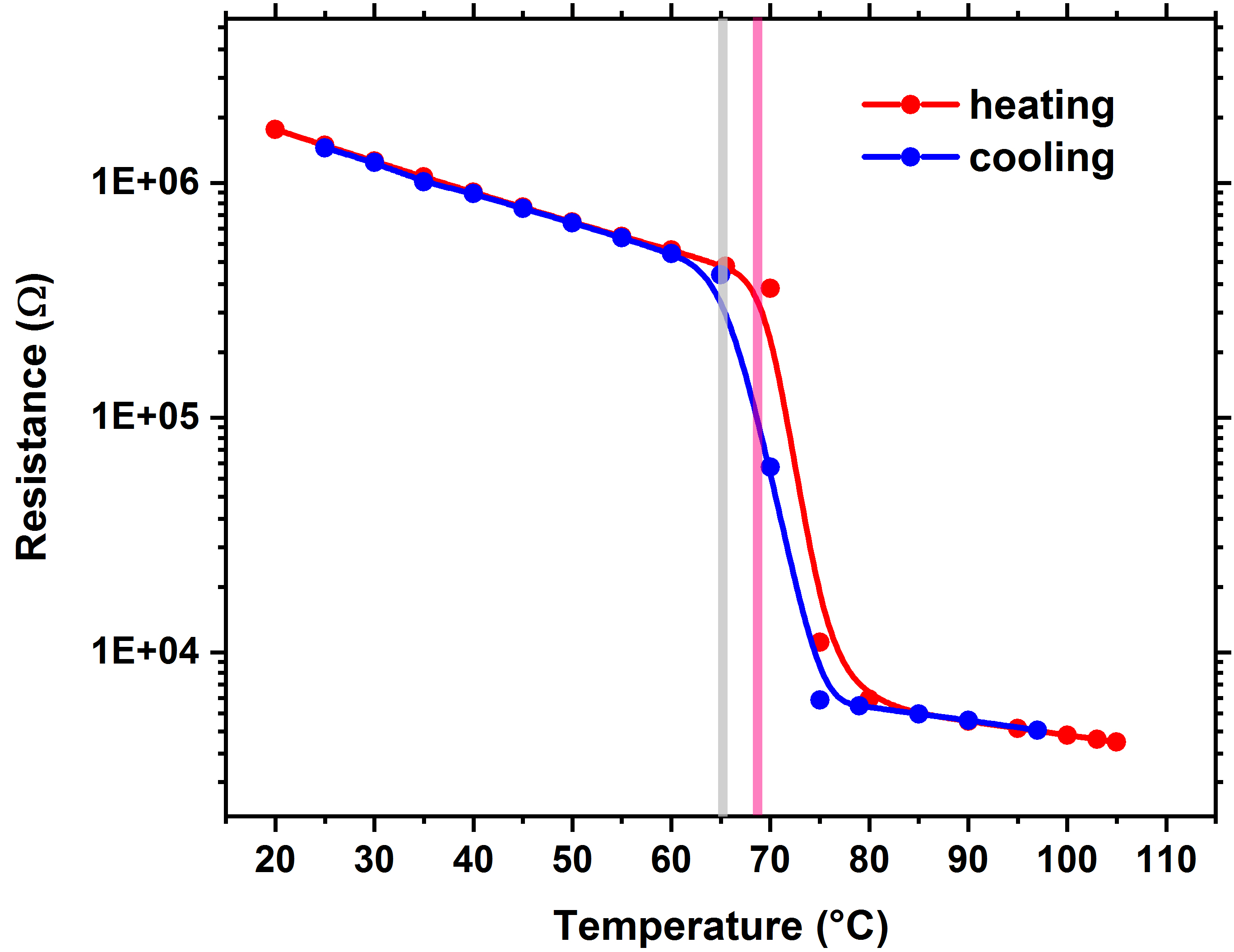


**Supplementary Figure S2 Variation with temperature of the overall resistance of the polarizer**. As the conductivity of the VO_2_ can be affected by the temperature, the whole resistance of the polarizer can be changed as temperature change. It can be found that the resistance of the polarizer decreases dramatically for temperatures higher than 71°C. Therefore, if the voltage is applied, the current flow through and heat the VO_2_ pattern. When the temperature reached to the threshold temperature, the whole resistance of the polarizer can be reduced, and MIT activation can be observed.


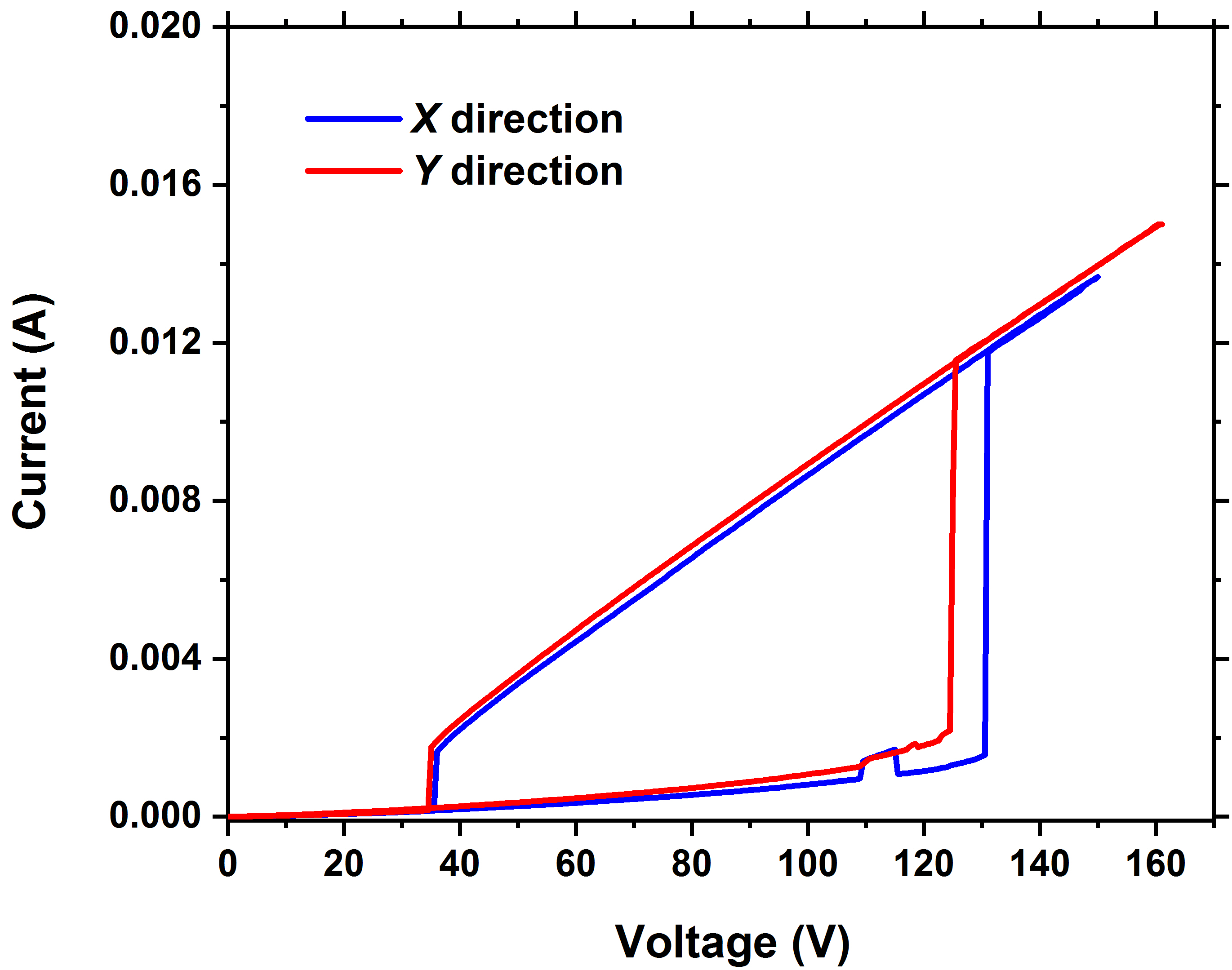


**Supplementary Figure S3 Superposed current-voltage (I-V) characteristics of the device, at room temperature, in *X*- and *Y*-directions (blue-, respectively red-curves).** It shows the onset of the MIT in the VO_2_ patterns as abrupt jumps in current for similar threshold voltages close to 130 V, which indicates that the threshold voltages from the insulating state into the metallic state are around 130V in *X*- and *Y*-directions. However, the threshold voltages from the metallic state into the insulating state are only 35V. This is because in metallic states, the resistance of the polarizer is much lower than that in insulating state. Therefore, a low supplied voltage can generate enough heating power to maintain the material in its metallic state. The behavior of threshold voltages and of the overall I-V hysteresis were extensively investigated in the literature and their characteristics are temperature-dependent (e.g. the higher the temperature, the lower the threshold voltages).

**Supplementary Figure S4 Measurement setup of the current-voltage characteristics of the device and of its response time.** A fabricated active polarizer was measured by time response analysis. Ch1 and Ch2 are connected to oscilloscope (Ch1 is the overall applied voltage, Ch2 is proportional to the current in the circuit).

**a.** **b.**

**Supplementary Figure S5 (a) Measured overall source applied voltage (Ch1, here with a magnitude of 100V) and response voltage across the device (Ch2) versus time (b) Response time versus the source applied voltage (Ch1) at different bias temperatures close to the transition temperature, before the onset of the MIT.** The square-type applied voltage from the voltage source (Ch1) varies from 0 to 200 V. When the applied voltage is lower than the device’s threshold voltage, the current in the circuit is small, which indicates that the device resistance is high, no MIT activation is observed. While the applied voltage is higher than or equal to threshold voltage, abrupt voltage jumps in Ch2 and abrupt drop in Ch1 is observed, which means that MIT activation of the VO_2_ patterns is occurred. The response time of the device (*τ_R_*) is determined by the magnitude of the applied voltage (Ch1) as well as the device’s temperature. The response time versus the applied voltage at different temperatures shows that the higher the applied voltage, the faster the response time of the device (*τ_R_*). For instance, at 65°C, the response time is 217 ms for 100V. However, when the applied voltage increases up to 200V, the response time is 16 ms Besides, for a given applied voltage, the response time can be shortened as temperature increases. For example, the response time is 16 ms at 65°C and 4.2 ms at 68°C for an applied voltage equal to 200 V. The modulation speed of the design is in the millisecond time scale, which is faster than approaches using mechanical stimuli (300ms as reported in [12]) although slower than optical-based solutions [13] [15] [18].


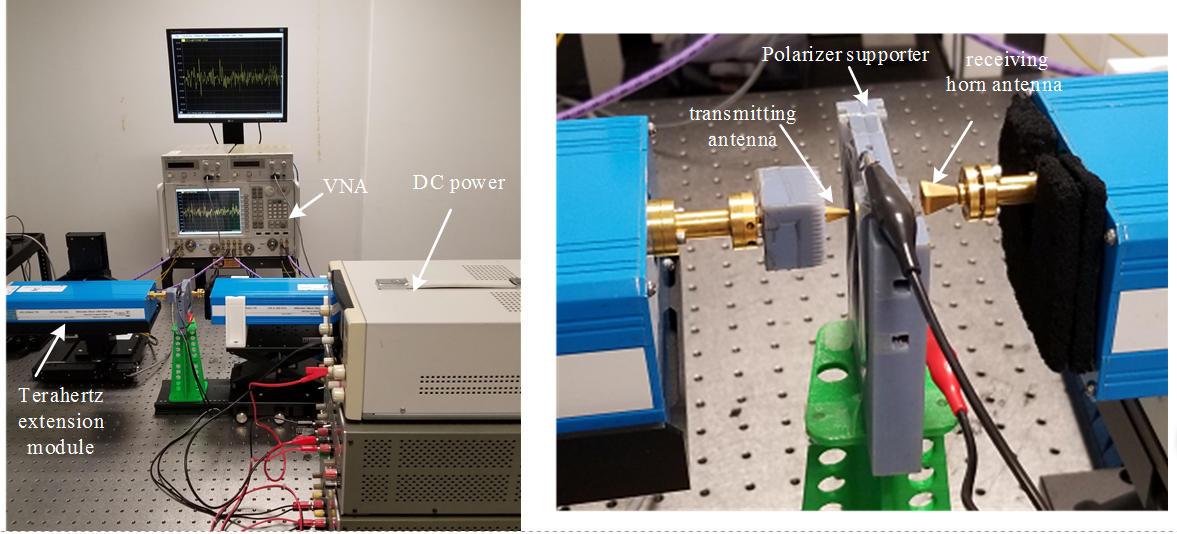


**Supplementary Figure S6** **Measurement setup of the EM characteristics of the device.** A pair of THz module (OML V02.2VNA2-T/R) are connected to the vector network analyzer (Agilent N5245A) to extend the operating frequency to 325–500 GHz. The transmitting antenna is an open waveguide which can generate a vertically polarized EM wave. While the receiving antenna is a standard horn, which has the same polarization with the transmitting antenna. The testing sample is inserted in the polarizer supporter.
